# Factors Associated with Physician Agreement on Verbal Autopsy of over 11500 Injury Deaths in India

**DOI:** 10.1371/journal.pone.0030336

**Published:** 2012-01-17

**Authors:** Marvin Hsiao, Shaun K. Morris, Diego G. Bassani, Ann L. Montgomery, J. S. Thakur, Prabhat Jha

**Affiliations:** 1 Centre for Global Health Research, Li Ka Shing Knowledge Institute, St. Michael's Hospital, University of Toronto, Toronto, Canada; 2 Department of Surgery, St. Michael's Hospital, University of Toronto, Toronto, Canada; 3 Division of Infectious Diseases, Department of Pediatrics, Hospital for Sick Children, University of Toronto, Toronto, Canada; 4 Dalla Lana School of Public Health, University of Toronto, Toronto, Canada; 5 Child Health and Evaluative Sciences, Hospital for Sick Children, Toronto, Canada; 6 Department of Community Medicine, Post Graduate Institute of Medical Education and Research (PGIMER), Chandigarh, India; University of Bochum, Germany

## Abstract

**Introduction:**

Worldwide, injuries account for 9.8% of all deaths. The majority of these deaths occur in low- and middle-income countries where vital registration systems are often inadequate. Verbal autopsy (VA) is a tool used to ascertain cause of death in such settings. Validation studies for VA using hospital diagnosed causes of death as comparisons have shown that injury deaths can be reliably diagnosed by VA. However, no study has assessed the factors that may affect physicians' abilities to code specific causes of injury death using VA.

**Method/Principal Findings:**

This study used data from over 11 500 verbal autopsies of injury deaths from the Million Death Study (MDS) in which 6.3 million people in India were monitored from 2001–2003 for vital events. Deaths that occurred in the MDS were coded by two independent physicians. This study focused on whether physician agreement on the classification of injury deaths was affected by characteristics of the deceased and respondent. Agreement was analyzed using three primary methods: 1) kappa statistic; 2) sensitivity and specificity analysis using the final VA diagnosed category of injury death as gold standard; and 3) multivariate logistic regression using a conceptual hierarchical model. The overall agreement for all injury deaths was 77.9% with a kappa of 0.74 (99% CI 0.74–0.75). Deaths in the injury categories of “transport”, “falls”, “drowning” and “other unintentional injury” occurring outside the home were associated with greater physician agreement than those occurring at home. In contrast, self-inflicted injury deaths that occurred outside the home were associated with lower physician agreement.

**Conclusions/Significance:**

With few exceptions, most characteristics of the deceased and the respondent did not influence physician agreement on the classification of injury deaths. Physician training and continued adaptation of the VA tool should focus on the reasons these factors influenced physician agreement.

## Introduction

Worldwide, injuries account for about 10% of all deaths and about 12% of the total burden of diseases measured in disability-adjusted life years [1]. The majority of these injuries occur in low and middle-income countries [1]. In such settings with limited resources, deaths often occur outside the formal healthcare system and are not recorded in the country's vital registration system. The lack of accurate vital statistical data hampers public health action, policy making, and the implementation of evidence-based interventions.

Verbal autopsy (VA) is a cost-effective tool for ascertaining cause of death in low-resource settings with incomplete vital registration [2]. The VA relies on the assumption that each cause of death has a unique set of signs and symptoms that can be used to distinguish between different causes of death. The VA also assumes that the signs and symptoms leading up to a death can be accurately reported by the deceased's family or acquaintances during a standardized interview.

Various methodologies exist to determine a cause of death based on the signs and symptoms collected on the VA questionnaire [3]. These include data- or expert-derived algorithms [4], symptom pattern methodology [5], and probabilistic models [6], [7]. Another common methodology utilizes two or more trained physicians to review the VA form to determine a cause of death [2].

Studies using VA with physician coders have been externally validated by comparing the VA cause of death to hospital-based registries or death certificates [8]–[13]. However, in low-resource settings, this method of validation may be biased because people with access to healthcare facilities, death certificates, or death registration are often not representative of the entire population.

Injury as a broad category of death can be diagnosed with high sensitivity, specificity, and positive predictive value by VA [11], [12], [14]. However, no study has assessed the factors that may influence the ability of physicians to distinguish between different injuries using VA. We used physician agreement as a metric in our studies to measure the performance characteristics of VA. Although no inference can be made on accuracy or external validity, higher physician agreement should be correlated with better reliability of VA assigned cause of death. We previously reported that younger age and female gender were associated with lower agreement between physicians on the cause of childhood deaths in India [15]. The purpose of our current study is to investigate whether characteristics of the respondent and deceased are associated with physician agreement on the classification of injury deaths.

## Methods

This study uses data from the Million Death Study (MDS); the methodology of the MDS is published in detail elsewhere [2]. In brief, the MDS monitored the vital statistics of 6.3 million people in 1.1 million nationally representative households in India from 2001–2003. A total of 6671 sampling units, each with an average of 150 households, were selected by stratified random sampling based on urban or rural setting, village or city population size, and female literacy rate according to the 1991 national census. For every death occurring in these households, a verbal autopsy (VA) questionnaire called RHIME (Routine, Reliable, Representative and Re-sampled Household Investigation of Mortality with Medical Evaluation; available at http://www.cghr.org/index.php/training/verbal-autopsy-forms-2/) was completed by a trained fieldworker based on a structured interview with a family member or close acquaintance of the deceased. RHIME includes both preset close-ended questions and an open-ended narrative. Each completed VA questionnaire was independently reviewed by two physicians trained to use the VA questionnaire to determine cause of death in World Health Organization International Classification of Disease 10 (ICD-10) code [16]. The physicians also provided a set of keywords that were important in determining the cause of death. If the two physicians initially agreed on the same cause of death, the corresponding ICD-10 code was assigned as the final cause of death. If the two physicians initially disagreed, they were required to anonymously reconcile by exchanging ICD-10 codes and keywords. If they agreed in this second reconciliation stage, the final cause of death was assigned. However, if there was still disagreement after the reconciliation stage, a third, senior physician adjudicated and assigned the final cause of death based on the VA questionnaire along with the ICD-10 codes and keywords provided by the first two physicians. A web-based system managed the coding process with more than 130,000 deaths coded by 130 physicians.

We used MDS data from 2001–2003 for this study. All deaths due to injury were grouped into 9 broad categories: transport injuries; falls; fire; drowning; venomous snakes, animals and plants; poisonings and other unintentional injuries; self-inflicted injuries; war, violence and other intentional injuries; and injuries of undetermined intent. The ICD-10 codes of these 9 injury categories are listed in Table 1, along with the number of deaths in each category stratified by age groups: children (≤14 years), adults(15 to 69 years), and elderly (≥70 years). For this study, physician agreement was defined as agreement of the first ICD-10 codes from the two physicians (i.e. the same injury category for both physicians at the first coding stage prior to the second reconciliation stage). The extent of agreement between two physicians beyond chance was assessed using the kappa statistic [17]. The 99% confidence interval for the kappa statistic was calculated using the bootstrap method for polychotomous variables with bias correction [18]. The Landis and Koch classification of inter-rater reliability was used to interpret the kappa statistic: ≤0.4 – poor to fair agreement; >0.4 to ≤0.6 – moderate agreement; >0.6 to ≤0.8 – substantial agreement; >0.8 – high agreement [17]. Analysis was conducted on the entire dataset and also stratified by age. Sensitivity and specificity of each physician's initial cause of death diagnosis was performed using the final RHIME cause of death diagnosis as the gold standard. This analysis should only be interpreted with the understanding that the gold standard (final cause of death diagnosis) was not independent of the test itself (initial physician cause of death diagnosis). The methodology used in this study to assess physician agreement and factors affecting agreement has been described in detail previously [15].

**Table 1:** Injury death category ICD-10 codes and numbers by age groups.

Injury Category	ICD-10 Codes	Number of Deaths		
		Children	Adults	Elderly
		(≤14 years)	(15–69 years)	(≥70 years)
Transport Injuries	V01-V99,Y85	239	1948	185
Falls	W00-W19	202	824	986
Fire	X00-X09	59	278	36
Drowning	W65-W74	456	406	42
Venomous Snakes, Animals and Plants	X20-X29,W57,W60	208	395	40
Other Unintentional Injuries	X40-X44,X46-X49, W20-W56,W58-W59,W64,W75-W99, X10-X19,X30-X39,X50-X52,X57-X59,Y40-Y84,Y86,Y88-Y89	323	1064	324
Self-Inflicted Injuries	X60-X84	53	2585	102
War, Violence and Other Intentional Injuries	X85-Y09,Y35-Y36,Y87	45	549	26
Injuries of Undetermined Intent	Y10-Y14,Y16-Y34,Y96-Y98	17	127	13

We used a multivariate logistic regression model to study the association between physician agreement and the geographic, socioeconomic, demographic, and other characteristics of the deceased and the respondent. The model was constructed using an *a priori* hierarchical conceptual framework that grouped variables into three blocks: distal, middle, and proximal [19]. The distal block included geographic factors (Empowered Action Group region including Assam (EAGA), geographic region, and urban or rural setting). The middle block included socio-demographic factors of the respondent (whether the respondent lived with the deceased, respondent's gender, education, religion, language, and relation to the deceased). The proximal block included characteristics of the deceased (deceased's gender, education, location of death, and whether the death was registered). Variable selection was conducted using backward elimination. A variable was retained in the final model if its likelihood ratio test resulted in p-value lower than 0.20. Respondents' education was predefined to remain in the final model regardless of p-value from the likelihood ratio test because of its expected influence on the quality of VA questionnaire. The final model for adults was also adjusted for the deceased's age as a continuous variable given the finding that increasing age was correlated linearly with lower physician agreement between injury death categories. Adjustment for age was not necessary in the children and elderly groups because the correlation between age and physician agreement within the group was not found to be significant. A factor was considered to be significantly associated with physician agreement if the likelihood ratio test resulted in p<0.05. All statistical analysis was performed using Stata SE version 10.0 [20].

Ethics approval for the MDS was obtained from the Postgraduate Institute of Medical Education and Research, Chandigarh, India and St. Michael's Hospital, Toronto, Ontario, Canada. Enrollment in the MDS was on a voluntary basis and the study posed no or minimal risks to enrolled subjects. Families were free to withdraw from the study. Verbal consent was obtained. All personal identifiers present in the raw data are anonymized before analysis. MDS data storage and usage protocols were described in detail elsewhere [2].

## Results

**Figure 1:**
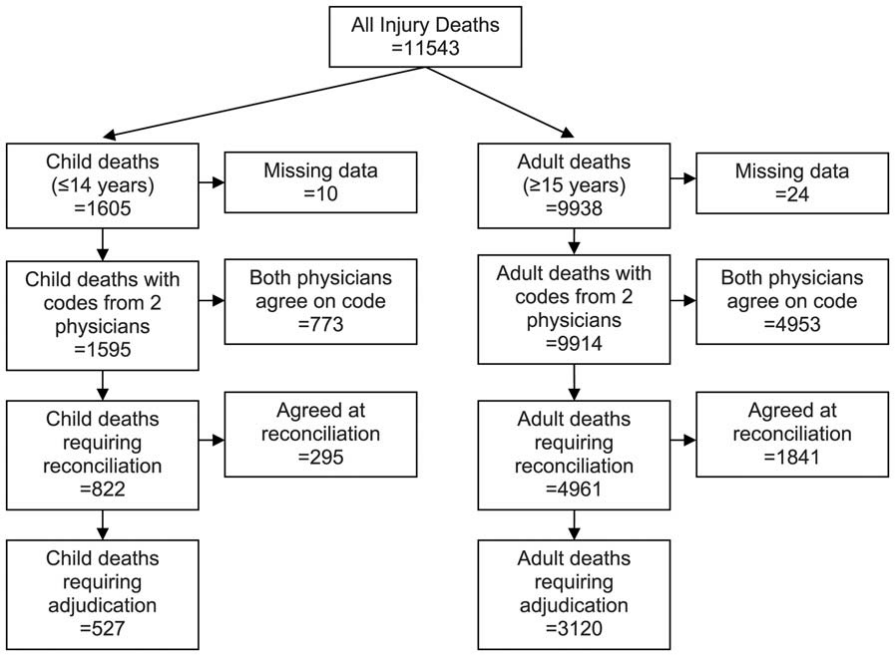
Flow diagram of Million Death Study injury deaths.

There were 11 543 injury deaths in the MDS from 2001–2003. Of these, 11 509 deaths (99.7%) were coded by two physicians and were included in this study. The process of determining the final cause of death is shown in Figure 1.

The summary of the kappa statistic analysis, stratified by age group, is shown in Table 2. For all age groups, non-overlapping 99% confidence intervals between one or more sub-categories of a predictor variable suggest statistical significance and require multivariate logistic regression modeling to rule out confounding effects. The overall agreement for all injury deaths was 77.9% with a kappa of 0.74 (99% CI 0.74–0.75). For children (≤14 years), there was 84.1% agreement and a kappa of 0.81 (99% CI 0.79–0.82). In this group, the 99% confidence intervals for kappa did not overlap between one or more sub-categories of the following variables: whether the respondent lived with the deceased, religion, language, deceased's gender, deceased's education, location of death, and whether the death was registered. For adults (15–69 years), there was 80.9% agreement overall and a kappa of 0.77 (99% CI 0.76–0.78). In this group, the 99% confidence intervals for kappa of all the variables did not overlap, except for respondent education and death registration. For the elderly (≥70 years), there was 58.2% agreement overall and a kappa of 0.44 (99% CI 0.40–0.45). In this age group, the variables where the 99% confidence interval for kappa did not overlap were whether the respondent lived with the deceased, respondent education, respondent relationship to the deceased, religion, language, deceased gender, location of death, and EAGA.

**Table 2:** Kappa statistic analysis of physician agreement for injury deaths by age group.

		Children (≤14 years)				Adults (15–69 years)				Elderly (≥70 years)			
Variable	Sub-Categories	n	% Agree	Kappa	99% CI	n	% Agree	Kappa	99% CI	n	% Agree	Kappa	99% CI
Overall		1595	84.1	0.81	0.79–0.82	8162	80.9	0.77	0.76–0.78	1752	58.2	0.44	0.40–0.45
Live with Status	Yes	1081	82.6	0.79	0.79–0.81*	4666	78.7	0.74	0.74–0.75*	1290	56.5	0.40	0.36–0.40*
	No	371	86.5	0.83	0.83–0.86*	2911	84.1	0.80	0.79–0.81*	349	61.0	0.51	0.50–0.53*
Respondent Gender	Male	894	83.8	0.81	0.79–0.84	4241	80.2	0.76	0.75–0.76*	950	58.4	0.44	0.41–0.45
	Female	665	84.5	0.81	0.80–0.82	3832	81.5	0.77	0.76–0.78*	788	57.7	0.43	0.41–0.47
Respondent Education	Less than Primary	908	83.5	0.80	0.78–0.82	3912	80.5	0.77	0.75–0.78	737	59.7	0.47	0.44–0.53*
	Primary	191	85.3	0.82	0.77–0.84	982	79.8	0.75	0.73–0.77	214	57.0	0.44	0.41–0.52*
	>Primary	466	84.6	0.82	0.80–0.83	3074	81.3	0.77	0.76–0.78	766	56.9	0.39	0.36–0.41*
Respondent Relationship	Related	1457	84.4	0.81	0.80–0.82	7346	80.6	0.77	0.76–0.77*	1603	57.4	0.42	0.40–0.45*
	Unrelated	95	85.3	0.82	0.80–0.85	699	83.6	0.79	0.77–0.79*	132	67.4	0.58	0.48–0.64*
Religion	Hindu	1271	84.1	0.81	0.80–0.82*	6786	81.2	0.77	0.76–0.78*	1427	58.7	0.44	0.44–0.47*
	Muslim	237	83.1	0.79	0.77–0.80*	675	80.4	0.77	0.73–0.80*	152	50.7	0.31	0.22–0.40*
	Other	83	86.8	0.84	0.78–0.90*	678	78.2	0.73	0.71–0.76*	166	59.6	0.46	0.39–0.52*
Language	Hindi	863	82.0	0.79	0.76–0.79*	2979	76.2	0.72	0.71–0.73*	646	54.3	0.37	0.33–0.39*
	English	68	95.6	0.95	0.90–0.98*	649	82.3	0.78	0.77–0.81*	98	67.4	0.58	0.53–0.70*
	Other	674	85.8	0.83	0.82–0.84*	4534	83.8	0.80	0.79–0.80*	1008	59.7	0.46	0.42–0.78*
Deceased Gender	Male	916	86.0	0.83	0.82–0.88*	5637	81.1	0.77	0.77–0.78*	901	59.4	0.48	0.47–0.50*
	Female	679	81.6	0.78	0.77–0.80*	2524	80.4	0.75	0.75–0.76*	851	56.9	0.38	0.37–0.40*
Deceased Education	Age-Appropriate	1337	84.7	0.82	0.81–0.82*	-	-	-	-	-	-	-	-
	Below Age-Appropriate	201	89.1	0.87	0.85–0.91*	-	-	-	-	-	-	-	-
	Less than Primary	-	-	-	-	3964	78.6	0.75	0.74–0.75*	1437	57.8	0.43	0.41–0.45
	Primary	-	-	-	-	1099	82.0	0.78	0.77–0.78*	133	60.2	0.48	0.40–0.52
	>Primary	-	-	-	-	2968	83.4	0.79	0.78–0.81*	156	58.3	0.42	0.39–0.50
Death Place	Home	685	80.7	0.77	0.76–0.78*	3188	77.2	0.70	0.70–0.72*	1348	53.0	0.33	0.31–0.35*
	Health Facility	248	81.1	0.78	0.76–0.81*	1652	80.8	0.77	0.76–0.77*	167	73.7	0.66	0.58–0.66*
	Other	578	89.6	0.86	0.84–0.87*	3041	84.9	0.81	0.80–0.81*	184	79.4	0.75	0.71–0.79*
Death Registration	Yes	401	86.8	0.84	0.82–0.85*	3409	81.3	0.77	0.76–0.79	693	56.1	0.39	0.36–0.40
	No	595	81.9	0.78	0.77–0.80*	1641	80.0	0.76	0.74–0.78	411	53.5	0.39	0.35–0.45
Rural/Urban	Rural	1429	84.3	0.81	0.79–0.82	6751	81.3	0.77	0.77–0.77*	1403	58.5	0.45	0.43–0.46
	Urban	166	82.5	0.79	0.69–0.86	1411	79.2	0.74	0.72–0.75*	349	56.7	0.37	0.31–0.46
EAG+Assam vs Non-EAG	EAGA	858	81.7	0.78	0.78–0.82	2840	77.5	0.74	0.73–0.75*	626	52.1	0.36	0.33–0.40*
	Non-EAGA	737	87.0	0.84	0.81–0.86	5322	82.7	0.78	0.78–0.79*	1126	61.6	0.48	0.45–0.52*

Footnote: * indicates that the 99% confidence intervals do not overlap between one or more sub categories of the variable. EAGA = Empowered Action Group including Assam.

The sensitivity and specificity of the initial physician coded cause of death was calculated using the final cause of death as gold standard (Table 3). There were 3 200 child, 16 346 adult, and 3 506 elderly initial physician coded cause of death (two codes per death). Excluding injuries of undetermined intent, the sensitivity of initial physician coding for all injury categories was above 82.0% in children, above 81.3% in adults, and above 72.7% in the elderly. Sensitivity for injuries of undetermined intent was the lowest in all three age groups – 61.8% in children, 59.7% in adults, and 53.8% in the elderly. The specificity of initial physician coding for all age groups was greater than 98.3% for all injury causes.

**Table 3:** Sensitivity and specificity of initial physician coded cause of death versus final RHIME cause.

Age Category	Cause of Death Category	n	Sensitivity	(99% CI)	Specificity	(99% CI)
Children	*Specific Causes*					
≤14 years	Drownings	911	96.8	(95.0–98.1)	99.1	(98.5–99.5)
	Transport Injuries	478	96.2	(93.4–98.1)	99.9	(99.5–100)
	Venomous Snakes, Animals, Plants	416	94.0	(90.3–96.6)	99.9	(99.5–100)
	Falls	403	89.1	(84.5–92.7)	99.1	(98.5–99.5)
	Fires	118	90.7	(81.6–96.2)	99.9	(99.6–100)
	Self-inflicted Injuries	106	89.6	(79.7–95.8)	99.6	(99.3–99.9)
	*Non-Specific Causes*					
	Other Unintentional Injuries	645	82.5	(78.3–86.2)	98.4	(97.6–99.0)
	War, Violence, and Other Intentional Injuries	89	82.0	(69.4–91.1)	99.8	(99.5–100)
	Injuries of Undetermined Intent	34	61.8	(38.5–81.7)	99.3	(98.9–99.6)
	*Total*	3200				
Adults	*Specific Causes*					
15–69 years	Self-inflicted Injuries	5169	92.9	(92.0–93.8)	99.5	(99.2–99.6)
	Transport Injuries	3895	94.9	(93.9–95.8)	99.5	(99.3–99.6)
	Falls	1648	82.0	(79.5–84.4)	99.2	(99.0–99.4)
	Drowning	812	90.1	(87.2–92.7)	99.7	(99.5–99.8)
	Venomous Snakes, Animals, Plants	790	93.3	(90.7–95.4)	99.9	(99.9–100)
	Fire	555	89.2	(85.4–92.3)	99.6	(99.5–99.7)
	*Non-Specific Causes*					
	Other Unintentional Injuries	2127	81.3	(79.1–83.5)	98.3	(98.1–98.6)
	War, Violence, and Other Intentional Injuries	1097	87.4	(84.6–89.9)	99.5	(99.3–99.6)
	Injuries of Undetermined Intent	253	59.7	(51.4–67.6)	99.1	(98.9–99.3)
	*Total*	16346				
Elderly	*Specific Causes*					
≥70 years	Falls	1971	75.3	(72.7–77.8)	98.5	(97.5–99.2)
	Transport Injuries	370	88.4	(83.4–92.3)	99.8	(99.5–99.9)
	Self-inflicted Injuries	204	94.6	(89.2–97.9)	99.9	(99.7–100)
	Drowning	84	83.3	(70.5–92.3)	100	(99.8–100)
	Venomous Snakes, Animals, Plants	80	86.3	(73.6–94.4)	99.9	(99.6–100)
	Fire	71	81.7	(67.2–91.8)	99.9	(99.6–100)
	*Non-Specific Causes*					
	Other Unintentional Injuries	648	72.7	(67.9–77.1)	97.9	(97.1–98.6)
	War, Violence, and Other Intentional Injuries	52	76.9	(58.9–89.9)	99.6	(99.3–99.8)
	Injuries of Undetermined Intent	26	53.8	(28.1–78.2)	99.8	(99.6–100)
	*Total*	3506				

The results of the multivariate logistic regression on factors influencing physician agreement are summarized in Table 4. For adults, physicians were more likely to agree on the classification of injury deaths if deaths occurred at a health facility (OR 1.19, 95% CI 1.01–1.39) and at a location outside of home or health facility (OR 1.50, 95% CI 1.30–1.73) compared to deaths at home. We postulated that injury deaths that are more likely to occur outside of home or health facility, such as transport injuries and drowning, may be easier to classify and would therefore have greater physician agreement. When we stratified the analysis by injury category, death location outside of home was associated with greater physician agreement only for deaths due to transport injury, drowning, and other unintentional injury (Table 5). In adult transport injury deaths, the odds ratio for physician agreement was 5.41 (95% CI 3.18–9.22) for deaths occurring at a health facility and 5.06 (95% CI 3.23–7.92) for deaths at a location outside of home or health facility. For both drowning and other unintentional injury deaths among adults, physician agreement was greater only for deaths that occurred at a location outside of the home or health facility (drowning - OR 2.07, 95% CI 1.08–3.99; other unintentional injury - OR 1.76, 95% CI 1.21–2.56) compared to deaths that occurred at home. In this age group, there was lower physician agreement for deaths due to self-inflicted injury that occurred at locations outside of the home or health facility (OR 0.60; 95% CI 0.44–0.81) compared to self-inflicted deaths at home.

**Table 4:** Multivariate logistic regression analysis of variables affecting physician agreement by age category.

		Children (≤14 years)				Adults (15–69 years)				Elderly (≥70 years)			
Variable	Sub-Categories	n	OR	(95% CI)	p	n	OR	(95% CI)	p	n	OR	(95% CI)	p
Live with	Yes	1089	1.00		0.1073	4676	1.00		0.0000	1290	1.00		0.7086
	No	372	1.32	(0.94–1.85)		2922	1.33	(1.18–1.51)		350	1.05	(0.80–1.39)	
Respondent Gender	Male	900	1.00		0.9581	4254	1.00		0.3577	952	1.00		0.2137
	Female	669	1.01	(0.74–1.37)		3841	1.06	(0.94–1.20)		788	0.88	(0.71–1.08)	
Respondent Education	Less than Primary	914	1.00		0.9796	3926	1.00		0.2256	738	1.00		0.1191
	Primary	191	0.99	(0.62–1.58)		983	0.90	(0.75–1.08)		214	0.77	(0.56–1.06)	
	>Primary	495	0.96	(0.58–1.58)		3222	1.06	(0.93–1.20)		794	0.82	(0.66–1.02)	
Respondent Relationship	Parent	895	1.00		0.2454	-	-	-	-	-	-	-	-
	Other Relative	571	1.28	(0.93–1.76)		-	-	-	-	-	-	-	-
	Neighbour	96	0.89	(0.47–1.70)		-	-	-	-	-	-	-	-
	Relative	-	-	-	-	7366	1.00		0.6258	1604	1.00		0.0312
	Neighbour	-	-	-	-	701	1.06	(0.84–1.34)		132	1.52	(1.03–2.23)	
Religion	Hindu	1280	1.00		0.7238	6804	1.00		0.3863	1429	1.00		0.1411
	Muslim	237	0.93	(0.62–1.40)		678	1.00	(0.81–1.23)		152	0.70	(0.49–1.00)	
	Other	84	1.30	(0.59–2.88)		679	0.86	(0.69–1.07)		166	0.93	(0.65–1.32)	
Language	Hindi	863	1.00		0.0899	3001	1.00		0.1040	648	1.00		0.8252
	English	68	3.46	(0.98–12.2)		649	1.03	(0.77–1.37)		98	0.92	(0.51–1.66)	
	Other	674	1.27	(0.76–2.12)		4534	1.21	(0.98–1.48)		1008	0.88	(0.59–1.32)	
Deceased Gender	Male	924	1.00		0.4518	5654	1.00		0.8721	903	1.00		0.7541
	Female	681	0.87	(0.61–1.25)		2529	1.01	(0.88–1.16)		851	0.96	(0.74–1.24)	
Deceased Education	Age-appropriate	1346	1.00		0.8820	-	-	-	-	-	-	-	-
	Below Age-appropriate	201	1.05	(0.57–1.92)		-	-	-	-	-	-	-	-
	Less than Primary	-	-	-	-	3976	1.00		0.4979	1438	1.00		0.9613
	Primary	-	-	-	-	1100	1.00	(0.83–1.22)		133	0.94	(0.58–1.51)	
	>Primary	-	-	-	-	2975	1.09	(0.94–1.27)		157	0.97	(0.60–1.57)	
Death Place	Home	692	1.00		0.0009	3195	1.00		0.0000	1350	1.00		0.0000
	Health Facility	250	0.98	(0.59–1.61)		1654	1.19	(1.01–1.39)		167	2.31	(1.58–3.36)	
	Other	579	2.18	(1.40–3.40)		3053	1.50	(1.30–1.73)		184	3.28	(2.22–4.83)	
Death Registration	No	595	1.00		0.1859	1650	1.00		0.7483	411	1.00		0.5202
	Yes	401	1.39	(0.85–2.28)		3419	0.97	(0.80–1.18)		695	0.90	(0.64–1.25)	

Footnote: Results are adjusted for region, EAGA, urban/rural and age.

**Table 5:** Place of death analysis of physician agreement stratified by injury category.

		Adults (15–69 years)			Elderly (≥70 years)		
Injury Category	Place of Death	n	OR	(95% CI)	n	OR	(95% CI)
Transport Injuries	Home	208	1		77	1	
	Health Facility	452	5.41*	(3.18–9.22)	43	4.92*	(1.55–15.69)
	Other	1231	5.06*	(3.23–7.92)	59	4.18*	(1.51–11.58)
Falls	Home	490	1		852	1	
	Health Facility	155	1.20	(0.77–1.86)	72	3.12*	(1.73–5.63)
	Other	151	1.49	(0.90–2.46)	36	2.42*	(1.13–5.15)
Fire	Home	96	1		24	1	
	Health Facility	137	1.39	(0.65–2.97)	7	-	-
	Other	39	1.29	(0.46–3.56)	4	-	-
Drowning	Home	104	1		17	1	
	Health Facility	15	3.74	(0.71–19.8)	0	-	-
	Other	266	2.07*	(1.08–3.99)	24	-	-
Venomous Snakes, Animals and Plants	Home	196	1		30	1	
	Health Facility	91	1.43	(0.57–3.56)	7	-	-
	Other	100	0.99	(0.44–2.22)	2	-	-
Other Unintentional Injuries	Home	483	1		260	1	
	Health Facility	213	0.79	(0.55–1.14)	25	0.58	(0.21–1.55)
	Other	333	1.76*	(1.21–2.56)	28	5.96*	(1.90–18.69)
Self-Inflicted Injuries	Home	1440	1		61	1	
	Health Facility	491	0.75	(0.53–1.05)	11	-	-
	Other	581	0.60*	(0.44–0.81)	24	-	-
War, Violence and Other Intentional Injuries	Home	135	1		19	1	
	Health Facility	72	0.66	(0.32–1.35)	1	-	-
	Other	301	1.02	(0.59–1.77)	6	-	-
Injuries of Undetermined Intent	Home	43	1		10	1	
	Health Facility	28	1.65	(0.34–8.00)	1	-	-
	Other	51	1.61	(0.43–6.08)	1	-	-

Footnote: * indicates statistical significance. - indicates insufficient number of deaths for regression analysis.

Also for adults, physicians were more likely to agree on the classification of injury deaths if the respondent did not live with the deceased (OR 1.33; 95% CI 1.18–1.51) compared to those who did live with the deceased. This association was similar in all injury categories (data not shown).

For the elderly, physician agreement was greater for deaths that occurred outside the home, whether at a health facility (OR 2.31, 95% CI 1.58–3.36) or at a location other than the home or health facility (OR 3.28, 95% CI 2.22–4.83). When stratified by injury category, greater physician agreement for elderly deaths that occurred outside the home was apparent only for transport injury, fall, and other unintentional injury deaths (Table 5). In transport injury deaths, physicians were more likely to agree for deaths that occurred at a health facility (OR 4.92, 95% CI 1.55–15.69) and at a location outside of the home or health facility (OR 4.18, 95% CI 1.51–11.58) compared to deaths that occurred at the home. Similarly in elderly deaths due to falls, physicians were more likely to agree for deaths that occurred at a health facility (OR 3.12, 95% CI 1.73–5.63) and at a location outside of the home or health facility (OR 2.42, 95% CI 1.13–5.15) compared to at the home. For other unintentional injury deaths in this age group, physician agreement was greater only for deaths that occurred outside of the home or health facility (OR 5.96, 95% CI 1.90–18.69) compared to at the home. Physicians were also more likely to agree on cause of deaths among the elderly if the respondent was a neighbor rather than a relative (OR 1.52, 95% CI 1.03–2.23). This association was similar among all injury categories.

For children, the only variable significantly associated with physician agreement was the location of death. Physician agreement was more common for deaths that occurred outside of the home or health facility (OR 2.18, 95% CI 1.40–3.40), regardless of the injury category.

## Discussion

The purpose of this analysis was to assess factors that may influence physicians' ability to correctly classify injury deaths based on VA questionnaires. We used physician agreement as the metric in our analysis under the assumption that poor agreement is correlated with difficulty in classifying injury deaths. Several validation studies have shown that VA tools perform well for injury deaths as one broad category with high sensitivity, specificity, and positive predictive value when compared to hospital records and death certificates [11], [12], [14]. However, to the best of our knowledge, no studies have assessed the factors that influence physician's ability to further classify injury deaths into specific categories using VA. From a public health injury prevention perspective, differentiating between injury categories is essential to priority setting and formulating targeted interventions. Using kappa statistic, sensitivity and specificity analysis, and multivariate logistic regression, this study specifically assessed factors that may influence physician agreement in injury deaths.

Using childhood deaths (age ≤14) of all causes, we previously reported that physician agreement was not affected by features of the death itself or by most geographic, socioeconomic, or demographic characteristics of the respondent and/or deceased. The exceptions were with the gender and age of the deceased [15]. This study of adult and child injury deaths also suggests that, with few exceptions, physician agreement on category of injury death was not affected by these factors. Specifically, we did not find consistent significant differences in physician agreement based on geographic factors, respondents' gender, education, religion, language, or on the gender, education, and death registration of the deceased. The similar level of agreement across all these variables is reassuring to all VA based studies.

Injury deaths are prevalent in the elderly population in India (manuscript in preparation). While the ability of VA to yield a broad classification of the underlying cause of death diminishes for deaths that occur over age 70 [2], we have shown that, even in the elderly population, VA performed reasonably well in distinguishing between different injury categories.

### Location of Death

For adult and elderly deaths in the categories of transport injury, other unintentional injury, fall (elderly only), and drowning (adults only), the likelihood of physician agreement increased if the location of death was outside of home. This association suggests that location of death provides an important clue for physicians in determining the cause of death for these four injury categories. One hypothesis is that for these categories, deaths occurring outside of home may be due to injuries of higher severity and acuity, resulting in a more immediate death that may be easier to classify. On the contrary, deaths occurring at home may have longer time lag between injury and death (for example, an elderly person becomes injured in a fall or car accident, undergoes treatment in hospital for several months, is eventually discharged and dies at home shortly after). When there is a longer lag time and opportunity for more intervening events between initial injury and death, as we hypothesize for injury deaths at home, physicians may have more difficulty determining cause of death. In contrast to these four categories, however, deaths due to self-inflicted injury were associated with lower physician agreement if the death occurred outside of home or health facility. One possible explanation for this finding is that the circumstances around a self-inflicted injury death is likely not as well known or reported by the respondent if the death occurred outside of home or health facility. On the other hand, detailed information on self-inflicted deaths may be more available if the death occurred at home or at health facilities. Additional studies exploring the open-ended narrative section of the VA may provide support for these hypotheses and generate additional explanations for the association between location of death and physician agreement.

In children, the association between increased physician agreement and death outside of home or health facility was also present. However, due to the smaller sample size we were unable to identify associations with particular injury categories. We believe that the association in children may be explained by the same rationale as adults, but the association will need to be assessed with a larger sample.

### Whether Respondent Lived with Deceased & Respondent Relationship

For adult deaths, physicians were more likely to agree on the classification of injury deaths if the respondent did not live with the deceased. Similarly for elderly deaths, physicians were more likely to agree if the respondent was a neighbour rather than a relative. We believe that this association may be reflective of the less detailed narratives given by the respondents who did not live with the deceased. For injury deaths, paradoxically, physicians may find narratives with fewer details easier to determine the cause of death. The less detailed narratives often comprise of only a few key words indicative of the cause of death such as “traffic accident” or “fall”. Detailed narratives, however, may describe multiple events during a longer intervening time gap between injury and death that, as hypothesized above for deaths occurring at home, may make determining cause of death more difficult.

### Limitations

Although we were able to assess the association between physician agreement and various characteristics of the respondent and deceased, we did not analyze the impact of factors related to the trained physician coders. Our analysis was performed under the assumption that the clinical experience, knowledge, medical specialty, and other factors that may affect the physician's ability to determine the cause of death based on VA were uniform among all physician coders. This assumption was likely not true. Nevertheless, we believe it is unlikely that this limitation would have led to a systematic bias in the results as the deaths were randomly assigned to the physician coders. Future studies should be performed to assess physician characteristics that affect their ability to determine cause of death using a VA instrument.

The sensitivity and specificity analysis of the cause of death determined in the first stage by physicians must be interpreted with the understanding that the gold standard used (RHIME-determined cause of death) was not independent from the initial physician assigned cause of death. Nonetheless, this analysis uncovered that physicians have more difficulty arriving at a diagnosis for deaths caused by injury of undetermined intent. Further interviewer and physician training to improve both the gathering and identification of details to arrive at a more specific category of injury death may help improve the VA tool.

The degree of physician agreement is contingent on the number of injury categories used in the analysis. Fewer categories would result in higher physician agreement and vice versa. We decided on the 9 injury categories based on a public health injury prevention perspective. Ideally, VA should be able to distinguish between injury categories such that the final output can be informative for public health priority setting, strategic planning, and disease monitoring. A limitation to our categorization is that no inference can be made on physician ability to diagnose specific causes of injury death within each injury category. Using transport injury deaths as example, the study cannot comment on whether VA is specific enough to determine the deceased's mode of transportation and the mechanism of collision (i.e. three character ICD-10 “V” codes). This limitation is even more important for the more heterogeneous injury categories of “other unintentional injuries” and “injuries of undetermined intent”.

In summary, physician agreement on the injury category cause of death was not affected by most characteristics of the deceased or respondent, with the exceptions of location of death, respondent relationship, and whether the respondent lived with the deceased. Specifically, for transport injury, fall, drowning, and other unintentional injury deaths, the location of death outside of the home was associated with greater physician agreement. In contrast, self-inflicted injury deaths that occurred outside of the home were associated with lower physician agreement. In addition, physicians were more likely to be in agreement if the respondent did not live with the deceased or if the respondent was a neighbour instead of a relative. Recognition of these factors that influence the physician's ability to determine cause of injury death is essential for continued adaptation and improvement of the VA tool.
