# Correction: Factors Associated with Physician Agreement on Verbal Autopsy of over 11500 Injury Deaths in India

**DOI:** 10.1371/annotation/7e655300-7920-4c75-b999-b2e32bfc2e8c

**Published:** 2013-11-08

**Authors:** Marvin Hsiao, Shaun K. Morris, Diego G. Bassani, Ann L. Montgomery, J. S. Thakur, Prabhat Jha

A funding organization was incorrectly omitted from the Funding statement. The Funding Statement should read: "This study was prepared with partial support from the Bill and Melinda Gates Foundation through the Disease Control Priorities Network at the University of Washington. The funders had no role in study design, data collection and analysis, decision to publish, or preparation of the manuscript." 

